# Patient involvement in patient safety: Protocol for developing an intervention using patient reports of organisational safety and patient incident reporting

**DOI:** 10.1186/1472-6963-11-130

**Published:** 2011-05-27

**Authors:** Jane K Ward, Rosemary RC McEachan, Rebecca Lawton, Gerry Armitage, Ian Watt, John Wright

**Affiliations:** 1 Bradford Institute for Health Research; Bradford Teaching Hospitals NHS Foundation Trust, Duckworth Lane, Bradford, BD9 6RJ, UK; 2 Institute of Psychological Sciences, University of Leeds, Leeds, LS2 9JT, UK; 3 School of Health Studies, University of Bradford, Trinity Road, Bradford BD5 0BB, UK; 4 Department of Health Sciences, University of York, York YO10 5DD, UK; 5 Hull York Medical School, John Hughlings Jackson Building, University of York, Heslington, York YO10 5DD, UK

## Abstract

**Background:**

Patients have the potential to provide a rich source of information on both organisational aspects of safety and patient safety incidents. This project aims to develop two patient safety interventions to promote organisational learning about safety - a patient measure of organisational safety (PMOS), and a patient incident reporting tool (PIRT) - to help the NHS prevent patient safety incidents by learning more about when and why they occur.

**Methods:**

To develop the PMOS 1) literature will be reviewed to identify similar measures and key contributory factors to error; 2) four patient focus groups will ascertain practicality and feasibility; 3) 25 patient interviews will elicit approximately 60 items across 10 domains; 4) 10 patient and clinician interviews will test acceptability and understanding. Qualitative data will be analysed using thematic content analysis.

To develop the PIRT 1) individual and then combined patient and clinician focus groups will provide guidance for the development of three potential reporting tools; 2) nine wards across three hospital directorates will pilot each of the tools for three months. The best performing tool will be identified from the frequency, volume and quality of reports.

The validity of both measures will be tested. 300 patients will be asked to complete the PMOS and PIRT during their stay in hospital. A sub-sample (N = 50) will complete the PMOS again one week later. Health professionals in participating wards will also be asked to complete the AHRQ safety culture questionnaire. Case notes for all patients will be reviewed. The psychometric properties of the PMOS will be assessed and a final valid and reliable version developed. Concurrent validity for the PIRT will be assessed by comparing reported incidents with those identified from case note review and the existing staff reporting scheme. In a subsequent study these tools will be used to provide information to wards/units about their priorities for patient safety. A patient panel will provide steering to the research.

**Discussion:**

The PMOS and PIRT aim to provide a reliable means of eliciting patient views about patient safety. Both interventions are likely to have relevance and practical utility for all NHS hospital trusts.

## 1. Background

The public expect safety to be a priority within health services. However, estimates show that as many as one in 10 patients are harmed while receiving hospital care [1-4]. Strategies to improve safety have focused on developing incident reporting systems, and changing systems of care and professional behaviour. However, there has recently been a growing interest in involving patients in safety initiatives. Indeed, patient involvement in safety orientated activities very much reflects recent UK government policy aims for people to be generally more involved in their care [5,6]. Internationally, patient involvement is also a key priority with the World Health Organisation's World Alliance for Patient Safety (WHO, WAPS) citing mobilisation and empowerment of patients as one of six action areas that will be taken forward in its 'Patients for Patient Safety' programme [7]. This approach advances the development and use of interventions to promote and support patients' (and their representatives) roles in securing their own safety in health care contexts. Patients are in a unique position to contribute to both learning about safety and improvements to the safety of health care systems, by feeding information about safety issues they have identified or experienced, into local and national safety reporting systems.

Despite international emphasis on patient involvement in safety there is a dearth of research evidence on the acceptability to patients and equivocal evidence to date that such involvement leads to improvements in safety. The evidence that exists indicates that patients are willing and able to participate in error prevention strategies [8] and have the potential to improve safety [9-12]. However, many factors hinder patient participation including acceptance of the new patient role, lack of medical knowledge, lack of confidence, co-morbidity and sociodemographic factors [13]. Thus, there is clearly a need to understand further how patients can best be involved and how they can act to improve safety of care.

Reason's well known model of organisational safety [14] states that organisational accidents are a result of a number of factors including active failures on the part of the individual (for example, attentional slips, or mistakes in decision making), and 'systems failures' encompassing latent failures (for example, budgeting or rostering descisions) and local working conditions (for example, equipment unavailable, ward or unit understaffed). These failures are often referred to as 'contributory factors'. Based on these ideas, measurement tools have been developed in high-risk industries to monitor organisations' 'safety health' [15,16]. However, currently no general means of assessing organisational safety or 'systems' failures exists within the NHS [although see a recent paper exploring this in relation to operating rooms and intensive care units: [17]]. Furthermore, no specific measures of organisational safety exist that ask for the views of customers or patients, despite patients being well placed to observe the organisation of their care and the practices around them. Scales measuring patients' perceptions of healthcare are available, for example measures of patient satisfaction [18-20] but these have been criticised for being subjective, unreliable and with little validity [21,22]. Therefore, there is a need for reliable and valid tools that allow patients the opportunity to provide feedback on the safety of their care environment to inform local and organisational changes to improve patient safety.

Learning from error is a key element of patient safety [23], and one way to learn is through the reporting and analysis of patient safety incidents. A patient safety incident (PSI) has been defined as "any unintended or unexpected incident which could have or did lead to harm for one or more patients receiving NHS care" [24]. This definition usefully encompasses a variety of situations relating to patient safety, across both adverse events themselves (e.g. medical, surgical or diagnostic error), and near misses (e.g. situations or processes which could have resulted in preventable harm to a patient, but were averted). Historically, efforts to learn from incident reports have been focused on staff-led reporting systems [25], with little attention paid to the potential of the patient as a valuable source of information about patient safety [11,26-29]. Indeed, it has been argued by some authors that the patient is uniquely placed to contribute to the quality and safety of their own care [30], with recent empirical work demonstrating the feasibility and value of patient reporting [for review: [31]; also [32-34]]. However, no study to date has attempted to systematically develop and evaluate the most effective method of patient reporting. In addition, no study has attempted to link reporting of patient safety incidents to mainstream quality improvement mechanisms.

The aim of the current study is to develop and test a patient measure of organisational safety and patient incident reporting tool which will be used separately, or in combination, to help the NHS respond and learn quickly from failures in organisational systems as well as patient safety incidents. Specific objectives related to the development of the two tools are as follows:

Developing the Patient Measure of Organisational Safety (PMOS)

1. To determine the most appropriate way of assessing patients perceptions of organisational safety (study 1)

2. To develop a draft PMOS using previous literature and additional qualitative interviews with patients (study 2)

3. To explore acceptability and understanding of the draft PMOS using semi-structured interviews with patients and health professionals (study 3).

Development of the Patient Incident Reporting Tool (PIRT)

4. Based on views of patients and health professionals, to develop 3 different mechanisms for capturing patient reports of patient safety incidents experienced whilst receiving treatment in hospital (study 4).

5. To identify which of the 3 mechanisms is a) most effective in generating reports and b) most acceptable to patients and health professionals (study 5).

Testing the PMOS and PIRT

6. To explore the effectiveness and reliability of the PMOS in detecting patient perceptions of organisational safety (study 6).

7. To compare the error incidence and quality of reports from the PIRT with other standard methods used in practice (case note review and the trust staff-led incident reporting system) (study 6).

## 2. Methods

**Figure 1:**
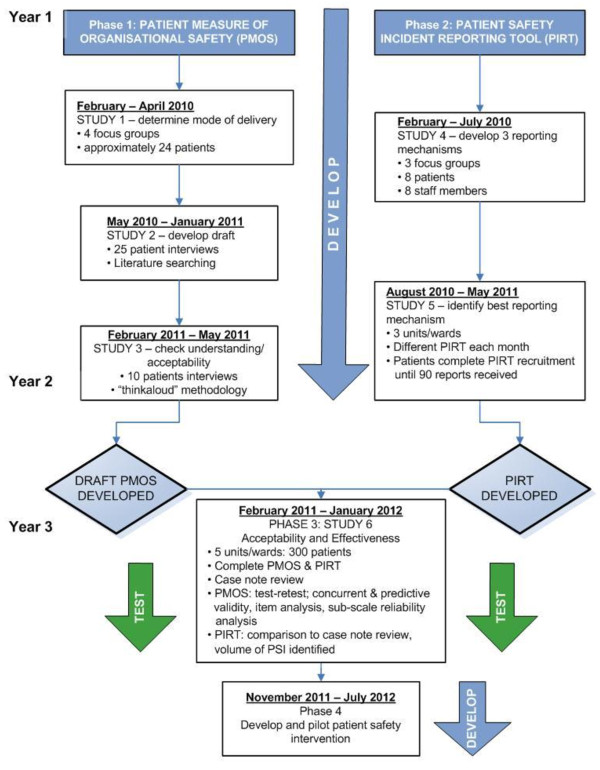
**Summary of research protocol**.

There are four phases to the project. The preparation of the protocols for each phase was done in collaboration with a variety of patients and health professionals at Bradford Teaching Hospitals NHS Foundation Trust. This was to ensure that the study aims and methods represented the views of both of these key stakeholder groups. In the first instance the development of the PMOS and PIRT will run in parallel, before the final versions of the tools are combined in a large quantitative study. The study protocol is summarised in Figure 1 has been approved by the Bradford Local Research Ethics Committee and Bradford Teaching Hospitals NHS Foundation Trust Research and Development department.

Due to the exploratory and developmental nature of the project, both qualitative and quantitative methods will be employed. Study participants will be patients (or parents and other carers of patients) and/or health professionals based at Bradford Teaching Hospitals NHS Foundation Trust, a large organisation encompassing two urban hospitals located in Bradford, United Kingdom. Bradford has an ethnically diverse ethnic community with approximately 19% of the population having South Asian origins, mainly from Pakistan [35].

### 2.1 Phase One: Developing a draft PMOS measure

#### 2.1.1. Objective 1: To determine the most appropriate way of assessing patients' perceptions of organisational safety

##### 2.1.1.1 Background

The first stage in developing the PMOS is to assess the feasibility of this novel measurement concept, with particular reference to the acceptability of patient identification of organisational safety issues.

##### 2.1.1.2 Method

Focus groups with patients will explore issues around the assessment of patients' perceptions of organisational safety.

##### 2.1.1.3 Sample and procedure

Four focus groups of 4-8 patients, relatives or carers will explore the following issues: a) the most viable method (e.g. questionnaire, structured diary) and medium (e.g. paper, web-based) for collecting patients' perceptions of organisational safety; b) when during the patient stay it would be best to collect these perceptions (e.g. during/at discharge); and c) face validity of a staff measure of organisational safety developed previously by the research team [36], modified to make appropriate for patients. Patients, and their relatives or carers, will be recruited in-situ from a variety of clinical settings.

##### 2.1.1.4 Analysis

Focus groups will be transcribed and analysed using content analysis [37].

#### 2.1.2 Objective 2: To develop a draft PMOS using previous literature and additional qualitative interviews with patients

##### 2.1.2.1 Background

Following assessment of the feasibility of the PMOS concept, a draft version of the measure will be constructed. Although no tools such as the PMOS currently exist, literature will be reviewed to identify any similar tools used in healthcare or other industries which may inform the development of the PMOS (e.g. the Consumer Assessment of Healthcare Providers and Systems Hospital Survey [19]; Hospital Survey on Patient Safety Culture [38]). Items will also be extracted from tools used in other high-hazard industries (e.g. Shell Tripod, Review: the Railtrack proactive error management tool). A systematic research review will identify key domains of systems' failures within hospital settings to ensure all appropriate contributory factors are measured within the tool. In addition a set of semi-structured interviews with patients will be used to construct additional items for inclusion within the measure.

##### 2.1.2.2 Method

Semi-structured interviews with patients will explore their experiences of safety within hospital, and what they might be in a position to identify.

##### 2.1.2.3 Sample and procedure

Interviews will be conducted using purposive sampling to include a broad population (e.g. across a range of ages, length of stay in hospital, degree of expertise in their condition, and ethnic groups). Interviews will proceed using an interview schedule until saturation of data is achieved (e.g. no new items are elicited) or 25 interviews are conducted. Following advice from an external steering group of patient safety researchers, policy makers and patient representatives, a decision was made to use two types of interviews to help identify items. Interviews will be conducted in either a 'bottom up' or 'top down' manner. In the former, patients/carers will be asked to describe a typical day of their most recent hospital experience. The interviewer will then identify possible systems failures, and probe to ascertain why the patient felt these occurred. In order to assist patients' focus on more organisational factors of care, the Systems Engineering Initiative for Patient Safety (SEIPS) model of work system and patient safety [39] will be presented. In the latter interviews the key domains of contributory factors identified from the systematic review will be used as a framework. Patients/carers will be asked how these factors may manifest themselves at a ward level.

##### 2.1.2.4 Analysis

All interviews will be transcribed. The first set of interviews will be analysed using thematic content analysis [40] to identify contributory factors that patients can spontaneously report. The second set of interviews using the contributory factor framework will be analysed using content analysis to explore a) what types of contributory factors patients feel they can identify, and b) how the contributory factors manifest themselves at a ward level. At this stage the authors envisage a pool in excess of 60 items for inclusion in version 1 of the measure.

#### 2.1.3 Objective 3: To explore the acceptability and understanding of the draft PMOS using semi-structured interviews with patients and health professionals

##### 2.1.3.1 Background

Having constructed the draft PMOS measure, it will be important to explore its acceptability and face validity. As the tool will ultimately be delivered at a ward/unit level, it is vital to ensure that it is acceptable to health professionals as well as patients.

##### 2.1.3.2 Method

Semi-structured interviews with patients and health professionals will explore the acceptability and understanding of the draft PMOS.

##### 2.1.3.3 Sample and procedure

Ten interviews will be conducted with health professionals and patients, purposively sampled from different care environments. These interviews will explore the face validity of the items, and to explore comprehension and interpretation. The interviews will also explore appropriate response options, for example whether participants find it easier to identify the presence or absence of a problem (answered with yes or no), or to have a scaled response (answered using a likert-type scale). Participants will use the 'think aloud' protocol [41] whilst completing the draft PMOS. The think aloud technique is a form of cognitive interviewing where participants are asked to verbalise all thoughts they have whilst completing a task (in this case completing the PMOS). Respondents are asked to 'talk aloud constantly' and to try not to plan or explain what they say. This process has been used successfully to understand comprehension of questionnaires in other domains [42].

##### 2.1.3.4 Analysis

A coding frame will be developed from inspection of two interviews. Responses to all questions will be coded by two independent reviewers to ascertain whether significant problems are apparent in the understanding or interpretation of the items. Disagreements will be discussed and resolved. Items which are problematic will be eliminated on the basis of these responses.

### 2.2 Phase two: Developing a patient incident reporting tool (PIRT)

#### 2.2.1 Objective 4: Based on views of patients and health professionals, to develop 3 different mechanisms for capturing patient reports of patient safety incidents experienced whilst receiving treatment in hospital

##### 2.2.1.1 Background

In order to ensure that any patient incident reporting tool we develop meets the perceived needs and expectations of both patients and health professionals, it will be important to work with both groups to generate possible mechanisms for capturing patient reports of patient safety incidents. This will also ensure that the final tool developed during the course of the research both reflects, and is embedded, in practice.

##### 2.2.1.2 Method

Three focus groups of 8-10 individuals will be conducted to discuss the feasibility and acceptability of patients reporting patient safety incidents within a hospital setting.

##### 2.2.1.3 Sample and procedure

The first focus group will comprise patients - or their relatives/carers - from across a range of recent health services experiences (e.g. acute through to chronic conditions; across the range of hospital specialties), with an appropriate demographic composition (e.g. age, gender, ethnic group). The feasibility of eliciting patient safety reports and acceptable methods for capturing such reports will be explored. The second focus group will be conducted with health professionals and managers from across a range of disciplines and medical specialties. Discussion will focus on the feasibility of the identified methods for eliciting patient reports in practice. The final focus group will bring together the patients and health professionals involved in groups 1 and 2, to further develop the suggested mechanisms and facilitate reaching a collective agreement in line with the study objective.

##### 2.2.1.4 Analysis

Outline ideas for three mechanisms will be agreed at the final focus group, by asking participants to identify their preferred three options from the suggestions presented at the earlier focus groups. In addition, a pragmatic qualitative analysis [43] will be undertaken on the transcripts of the focus groups, to explore the practice and patient context, and inform the subsequent development and implementation of the three mechanisms.

#### 2.2.2 Objective 5: To explore the efficacy of the 3 potential PIRT mechanisms

##### 2.2.2.1 Background

Having identified three mechanisms for capturing patient reports of patient safety incidents, we will then pilot these mechanisms to explore the quality of information they provide and their usage by patients.

##### 2.2.2.2 Method

Pilot study with randomisation of PIRT mechanisms at a ward/unit level.

##### 2.2.2.3 Sample and procedure

Nine wards across three directorates within the trust will act as hosts for the trial. Directorates selected to host the trial will represent different clinical specialties to ensure that the final mechanism is not context dependent, and can generalise across the hospital setting. Selection of the three wards within each host directorate will be done to minimise differences in terms of the type and demographics of the patients treated, to reduce threats to internal validity. Within each directorate, each of the three wards will trial one of the three draft incident reporting mechanisms, for a three month period, with the mechanisms randomly assigned to the three wards to reduce threats to external validity and recruitment bias [44]. During the study period, patients on each of the wards will be recruited and asked to provide reports of patient safety incidents via their designated mechanism. Recruitment will continue for three months with a view to collecting 90 patient safety reports - 30 for each of the three mechanisms. A similar study found 27.2 patient safety incidents were reported for every 100 patients [34]. Therefore it is anticipated we will need to recruit approximately 330 patients to this study to gain the required number of reports.

Where the mechanism requires non-written forms of report, these will be digitally recorded for transcription, or recorded using field notes, depending on patient preference. All reports received via each of the three draft reporting mechanisms will be reviewed regularly through the study period, to identify those that represent a PSI. Only reports agreed by the research team as fitting with this definition will be counted as a PSI report, and included as part of the ultimate PSI report total. In order to meet our duty of care to participants, health professionals and the Trust, we will collaborate with health professionals to develop a mechanism for timely identification of PSIs that require urgent action. Clinical members of the research team will then review PSI reports to assess whether there is a need for urgent action, and if so, will refer the incident to the Risk Management Team.

##### 2.2.2.4 Analysis

Volume of reports: The number of patients required to generate 30 reports will be used as a measure of the capacity of each mechanism to identify a PSI.

Quality of reports: Using a coding frame developed from a sample of 10% of received PSI reports, three independent raters (including health professionals and patient representatives) will independently code the data for PSI type, causation and potential outcome [43]. This will allow calculation of inter-rater reliability and will also assess the feasibility of the reporting and analysis process for practitioners. Following an assessment of the volume of reports generated, and quality of information achieved by each of the mechanisms, the best 'performing' mechanism will be chosen to be developed further.

### 2.3 Phase 3: To test the reliability and validity of the PMOS and to compare the error incidence and quality of patient elicited information from the PIRT to case note review and current incident reporting data

At this point the PMOS and PIRT projects will merge and will cover the following two objectives in study 6.

#### 2.3.1 Objectives 6 & 7: To explore the reliability and validity of the PMOS in detecting patients' perceptions of organisational safety; To compare the error incidence and quality of reports from the PIRT with other standard methods of error detection

##### 2.3.1.1 Background

Following the development of draft PMOS and PIRT measurement tools, a large scale study will undertake to establish the psychometric properties of both tools. In addition, consideration will be given to the nature of the data elicited from each tool, and any relationships between them. In doing so, we will begin to understand how these tools might work together as part of a wider patient safety intervention.

##### 2.3.1.2 Method

Validation study.

##### 2.3.1.3 Sample

Part A: 300 patients, or parents/carers of patients from a variety of units/wards will be recruited over a two month period. Allowing for 30% participation we will initially invite 900 patients to participant to provide sufficient responses to test the factor structure of the PMOS (minimum of 5 respondents per item [45]). For analysis pertaining to the PIRT, a sample size of 228 has been used successfully elsewhere to compare patient-reported events to those reported in the case notes and hospital reporting scheme [34].

Part B: Health professionals from participating wards (approximately N = 250) will complete the Hospital survey on patient safety culture (AHRQ) [38] adapted for UK hospitals.

##### 2.3.1.4 Procedure

Part A: Consenting patients will be asked to complete the PIRT and PMOS at some point in their stay (informed by earlier developmental work) and will have their medical notes reviewed after discharge. The exact timing of recruitment and when they will be asked to complete the PIRT and PMOS will be subject to the results from the earlier studies (for example, whether they should complete the PMOS/PIRT during their stay or at discharge, whether it is viable for patients to complete either tool retrospectively). One week after completion, a sub-sample of 50 respondents will be asked to complete the PMOS again to allow calculation of test-retest reliability. Responses to the PMOS and PIRT will be matched using an anonymous code. Upon discharge the case notes of the patient will be reviewed by a multidisciplinary team of health professionals and researchers using an explicit review method to detect adverse events [1-3,46].

Part B: Prior to the commencement of the study in all participating units, health professionals will be asked to complete the AHRQ hospital survey on patient safety culture [38]. No personally identifiable information will be collected although they will be asked to indicate the unit/department where they are employed. Health professionals will be asked to mail these questionnaires back to the research team via the internal mail system.

##### 2.3.1.5 Analysis

PMOS: To analyse the reliability and validity of the PMOS a number of statistical procedures will be conducted. First, item analysis and sub-scale analysis will assess the reliability of the scales. Test-retest reliability will also be assessed by correlations between PMOS scores at the two time points. Factor analysis will explore the dimensionality of the measure to assess whether clear distinctions between the different contributory factor domains can be identified. To measure concurrent validity the ability of the PMOS to differentiate different units/wards based on differences in the AHRQ hospital survey on patient safety will be assessed. Finally to assess predictive validity correlations between the PMOS scores and safety incident data (as identified from the PIRT) will be explored.

PIRT: Three main outcome measures will be explored: a) the frequency and volume of PSI reports; b) the quality of PSI reports; and c) the comparability of PSI reports with other review methods, to establish concurrent validity.

a) Frequency and volume of PSI reports

The frequency (number of reports) and volume (number of reports per participant) of PSIs will be compared with the frequency and volume of PSIs identified through case note review and from the hospitals standard incident reporting scheme, for those patients participating in the study. The case note review will adapt the explicit review method to detect adverse events [3] and incorporate methods used elsewhere to compare patient-reported adverse events to those identified in case note review [47,48].

In stage 1 of the review method, case notes will be screened by two nurses to identify potential incidents using 18 established and predefined criteria [3] modified to take account of what patients perceive as safety incidents [34]. In stage 2, two clinicians will review any records in which a PSI was identified in stage 1. A structured form will be used to determine a) whether the incident had potential for harm (either adverse event or near miss), b) impact, c) domain, d) cause and e) prevention, following guidelines from existing taxonomies [49]. In addition a standardised 6-point scale will be used to determine the role of clinical management rather than the disease process in occurrence of the patient safety incident. 10% of case notes will be reviewed by a second clinician to gather data on inter-rater reliability [50,51].

Staff incident reporting databases at the Trust allow identification by patient name. Thus any PSI reports pertaining to the sample of 300 patients in the study will also be collected. These reports will be anonymised and linked to patients' reports and case notes using a unique code. The frequency and volume of incidents recorded through the three channels (patient incident reporting, staff incident reporting and case note review) will be compared.

b) Quality of PSI reports

In order to assess the quality and richness of information contained within the reported incidents (e.g. to provide adequate information for organisational learning) two independent reviewers will rate the quality of the text from a random sample of 10% of incidents from all channels using previously validated techniques [43].

c) Comparability of the PSI reports with other review methods

To explore the concurrent validity of the PIRT data (e.g. is it picking up the same reports made by health professionals in case notes?), 40 case notes and patient safety reports will be coded together by two clinicians. Disagreements will be resolved by consensus.

### 2.4 Phase 4: Developing an Intervention

Once the PMOS and PIRT have been finalised we will explore ways of using the information obtained through these measures to develop an intervention aimed at fostering feedback and promoting organisational learning. The process will be facilitated by stakeholder feedback and involvement, for example through patient and health professionals' panels. At the end of this process we will apply for further approval to pilot the intervention in one trust, and then roll out to five other NHS trusts.

### 2.5 Patient Panel

A panel of patient representatives will provide direction and steering to the research throughout the course of the project via regular attendance at project meetings with the research, and at special bi-annual patient panel meetings.

## 3. Discussion

This paper has presented details of a large-scale applied research project, aimed at systematically developing a patient-centred patient safety intervention. The focus of this work is to create tools which can be used alone, or in combination, to shed light on the 'patients' view' of patient safety, and to allow health service organisations to make improvements using this important perspective on patient safety. The authors very much acknowledge the need to create tools which are usable by patients and health professionals, yet have utility in delivering meaningful information regarding patient safety outcomes across the range of clinical specialties that make up a hospital setting. Indeed, one of the key strengths of the design is the central role of patients/carers and health professionals across all stages of the research project, not just as participants, but as real collaborators in the development of the intervention. The authors firmly believe that using this partnership approach will ensure that the tools we develop reflect the realities of the patient and health professionals' experience, which is crucial if, going forward, the tools are to become embedded in practice.

Clearly, as with all patient safety research there are challenges as well as opportunities. It is anticipated that the main issues facing this research project fall into two main categories: engaging patients and engaging health professionals.

### Engaging patients

Although it has been demonstrated that patients are concerned about their safety whilst receiving treatment [33,34], it is unclear that this concern represents a willingness to engage in patient safety related activities [29]. Indeed, the willingness of patients to engage in patient safety research varies across a number of indices [52,53], and with most patient safety campaigns seemingly based on the assumption that patients will uniformly engage in safety activities, this presents researchers with a challenge: how to engage patients in organisation-level initiatives when their success may depend on individual-level factors? We also need to be mindful of the risk of patients feeling that we are shifting the weight of responsibility onto them, with the commensurate concern of receiving substandard or reduced care if they do not get involved [9]. In the light of all of the above issues, recruitment into the studies probably represents the most significant challenge to the success of the project.

Furthermore, effort needs to be directed at recruiting and engaging patients from all ethnicities and cultures. This is particularly important given the diverse nature of the local population in which the current project is located (Bradford, UK), with over-representation of certain ethnic groups in some units within the Trust hospitals. There is also an empirical imperative given the emerging evidence suggesting that non-native speaking patients may experience increased adverse events [54] but be less likely to report problems with their care [55]. Indeed, it has recently been suggested that "if patient safety engagement/partnership programs are to perform well in cross-cultural health care contexts, they need to be....appropriately informed by the perspectives and experiences of patients and families/nominated carers from minority cultural and language backgrounds" (p.1: [56]).

### Engaging health professionals

Two recent reviews of patient involvement in patient safety initiatives have concluded that health professionals have an important role in engaging patients in such initiatives [13,29]. Patients have been found to be far more willing to ask health professionals challenging questions if instructed by medical staff [52], and where health professionals consistently engage patients in discussions about their care as part of a trusting patient-professional relationship [53]. This underlines the importance of gaining the support of health professionals when introducing patient safety initiatives or interventions. Indeed, if patient engagement in safety is to succeed it is vital for health professionals to move beyond recognising the benefits of patient involvement, towards actively encouraging safety related behaviours in patients [28,53]. Our challenge therefore, is to ensure that our research proceeds in a collaborative way with health professionals as well as patients, to represent and integrate their views and concerns as much as possible into the design of the patient safety intervention. Looking ahead to the possible uptake of the tools across health services, it will be important to more fully understand the nature of health professionals' concerns about patient involvement [53], and ensure that interventions remain a collaboration between patients, health professionals and researchers, if they are to prove an effective means of proactively managing patient safety in the future.

Involving patients in their own safety is both a promising and policy-driven area for study, with the potential for delivering real changes to patient safety outcomes in the short- and longer-term. Development of the tools described here, using a partnership model encouraging real collaboration between patients, health professionals and researchers will help to ensure that the final intervention reflects the realities of the environment in which it will ultimately exist, increasing the likelihood of acceptance and use by patients in the future. The authors hope ultimately to demonstrate that these tools can provide a basis for services to understand more about patient safety in their areas, allowing them to achieve substantive improvement to patient safety outcomes.

## 4. Competing interests

The authors declare that they have no competing interests.

## 5. Authors' contributions

JKW and RRCM prepared the manuscript and manage the two projects described in this protocol. RL and GA are the project leads and creators of the original protocol outline. JW and IW are the chief investigators on the two projects. All authors read and approved the final manuscript.

## 6. Authors' information

All authors are based within the Yorkshire Quality and Safety Research Group, which represents a multi-disciplinary collaboration across the Bradford health care economy, other regional NHS trusts and the universities of Leeds, Bradford, York and Newcastle. The group leads a number of large applied health service research projects, and currently holds two NIHR programme grants in patient safety. Our research brings together health professionals, patients, managers and researchers, to develop evidence-based improvements to the quality and safety of health care.

## Pre-publication history

The pre-publication history for this paper can be accessed here:

http://www.biomedcentral.com/1472-6963/11/130/prepub
